# Subcellular Localization of GIGANTEA Regulates the Timing of Leaf Senescence and Flowering in *Arabidopsis*

**DOI:** 10.3389/fpls.2020.589707

**Published:** 2020-11-19

**Authors:** Hyunmin Kim, Su Jin Park, Yumi Kim, Hong Gil Nam

**Affiliations:** ^1^Center for Plant Aging Research, Institute for Basic Science, Daegu, South Korea; ^2^New Biology, Daegu Gyeongbuk Institute of Science & Technology, Daegu, South Korea

**Keywords:** circadian clock, leaf senescence, GIGANTEA, EARLY FLOWERING 4, subcellular localization, ORESARA 1, *Arabidopsis*

## Abstract

Plants undergo several important developmental transitions including flowering and senescence during their life cycle. Timing these transitions according to the environmental conditions increases plant fitness and productivity. The circadian clock senses various environmental cycles, including photoperiod, and synchronizes plant physiological processes to maximize plant fitness. Here, we propose that the cellular localization of GIGANTEA (GI), a key clock component, regulates leaf senescence and flowering in *Arabidopsis thaliana*. We show that GI, which connects the circadian clock with photoperiod-regulated flowering, induces leaf senescence depending on its subcellular localization. Overexpression of GI in the *gi* mutant rescued its delayed senescence phenotype but only when the GI protein was targeted to the nucleus, not when it was targeted to the cytosol. In the nucleus, EARLY FLOWERING 4 (ELF4) inhibited the binding of GI to *ORESARA 1* (*ORE1)* promoter to regulate leaf senescence. GI also positively regulated the day-peak of *ORE1* expression. These results indicate that like flowering, leaf senescence is also controlled by the location of GI in the cell. Taken together, our results suggest that ELF4 and GI act together to control flowering and senescence in *Arabidopsis*.

## Introduction

Plants undergo important developmental events, such as flowering and senescence, during their life cycle (Huijser and Schmid, 2011). Leaves are the main photosynthetic organs of a plant. During the growth period, photosynthetic products are stored in young leaves; however, at the senescence stage, these products are transferred to newly developing leaves or seeds (Woo et al., 2019). Thus, proper timing of developmental events is critical for maximizing plant fitness, and this timing is determined by plants through sensing the surrounding environmental conditions.

The endogenous biological clock, also known as the circadian clock, regulates almost all developmental processes in plants, ranging from germination to plant maturation, by sensing the environmental conditions. For example, the circadian clock regulates flowering by sensing the photoperiod (de Montaigu et al., 2010). One of the circadian clock components, *GIGANTEA* (*GI*), regulates photoperiod-dependent flowering in *Arabidopsis thaliana*. GI directly binds to the promoter of *CONSTANS* (*CO*), a key flowering regulator, and activates *CO* expression. However, GI promotes *CO* expression, enabling it to reach peak expression levels, only under long-day (LD) photoperiod, not under short-day (SD) photoperiod. This coincidence between the environmental condition (LDs) and internal rhythm (circadian rhythm) promotes flowering in *Arabidopsis*, and is known as the external coincidence model (Sawa et al., 2008). Interestingly, the control of GI on flowering time depends on the location of GI in the cell. The GI protein localizes to both the nucleus and the cytoplasm; however, cytosolic GI does not affect flowering time. GI must be transferred to the nucleus for activating *CO* expression (Kim et al., 2013a). In the nucleus, another clock component, EARLY FLOWERING 4 (ELF4), physically interacts with and sequesters the GI protein, which inhibits GI from binding to the *CO* promoter (Kim et al., 2013b). Thus, the subcellular localization of GI is important for regulating flowering time in *Arabidopsis*.

Recently, several studies showed that circadian clock components control leaf senescence in *Arabidopsis.* Leaf senescence is a critical developmental process involving the transition from nutrient accumulation to nutrient transfer to newly developing organs. ORESARA 1 (ORE1), a key positive regulator of senescence, is under the control of the circadian clock and is regulated by several clock components, such as Circadian Clock Associated 1 (CCA1; Song et al., 2018), Pseudo Response Regulator 9 (PRR9; Kim et al., 2018), and the evening complex [EC; composed of ELF3, ELF4, and LUX ARRYTHMO (LUX); Zhang et al., 2018]. GI, a key component of the circadian clock that is important for flowering, is also involved in the response to oxidative stress. *gi* mutants exhibit tolerance to hydrogen peroxide and extended plant longevity (Kurepa et al., 1998). These studies suggest that the perception of environmental conditions by the circadian clock is important for regulating the timing of leaf senescence. Previously, we reported that almost all clock mutants of *Arabidopsis* exhibit an early or a delayed senescence phenotype. Additionally, clock mutants show a strong correlation between flowering and leaf senescence phenotypes (Kim et al., 2018).

Here, we show that GI controls leaf senescence, similar to flowering time, in a location-dependent manner in *Arabidopsis*. Unlike the cytosol-localized GI, the nuclear-localized GI induces leaf senescence, suggesting that plants utilize the same regulatory mechanism for regulating two major developmental events: flowering and senescence. In the nucleus, ELF4 directly interacts with GI and inhibits its binding to the *ORE1* promoter. Taken together, these results suggest that GI acts as a mediator to couple flowering with leaf senescence for enhancing plant fitness and productivity.

## Materials and Methods

### Plant Materials and Growth Conditions

*Arabidopsis thaliana* ecotype Columbia (Col-0) was used as the wild-type in all experiments. *Arabidopsis thaliana* mutants, *gi-2* (Park et al., 1999), *gi-ko* (Martin-Tryon et al., 2007), *elf4-209* (Kolmos et al., 2009), and *gi-2 elf4-209* (Kim et al., 2012), and overexpression lines, *CsV:GI-GFP*, *elf4 CsV:GI-GFP*, and *VGE:GI-GFP* (Kim et al., 2013b), used in this study have been described previously. The *CsV:GI-GFP-NLS* and *CsV:GI-GFP-NES* vectors were constructed using the nuclear localization signal (NLS) (Kalderon et al., 1984) from SV4012 and nuclear export signal (NES) (Henderson and Eleftheriou, 2000) from MAPKII in the pPZP221 backbone (Hajdukiewicz et al., 1994). The *CsV:GI-GFP-NLS* and *CsV:GI-GFP-NES* constructs were introduced into the Col-0 line, and the *gi-2* mutation was then introduced via a genetic cross.

Plants were grown in an environmentally controlled growth room at 22°C under a LD (16 h light/8 h dark) or SD (8 h light/16 h dark) photoperiod and 100 μmol ⋅ m^–2^ ⋅ s^–1^ light intensity using fluorescent lamps. All physiological experiments were carried out using the third and fourth rosette leaves of plants. Leaf samples were obtained by cutting the leaves at approximately the middle of the petiole using a sharp scalpel to minimize wounding stress.

### Leaf Senescence Assay

Chlorophyll was extracted from individual leaves by heating in 95% ethanol at 85°C for 10 min. The chlorophyll concentration per fresh weight of leaf tissue was calculated as described previously (Lichtenthaler, 1987). The ratio of variable fluorescence to maximum fluorescence (Fv/Fm) was used as a measure of the photochemical efficiency of PSII (Oh et al., 1997), which was measured using an Imaging-PAM chlorophyll fluorometer (Heinz Walz GmbH, Effeltrich, Germany). Six biological replicates were performed.

### Gene Expression Analysis

Total RNA was extracted from leaves using WelPrep (Welgene, Daegu, Korea) and then treated with DNase I (Ambion, Austin, TX, United States) to remove any traces of contaminating DNA. Subsequently, 0.75 μg of DNase I-treated total RNA was reverse-transcribed using the ImProm II reverse transcriptase (Promega, Madison, WI, United States). The quantity of transcript in each sample was measured using real-time PCR on a CFX96 Touch^TM^ Real-Time PCR Detection System (Bio-Rad, Hercules, CA, United States) using universal SYBR Supermix (Bio-rad, Hercules, CA, United States). Fold changes in gene expression were calculated using the comparative threshold (CT) method and then normalized relative to the expression of *Actin2* (*ACT2*; At3g18780). Three biological replicates were performed. Primers used in this study are listed in Supplementary Table 1.

### Immunoblot Analysis

Immunoblot analyses of GI, HSP90, and H3 were performed as previously reported (Kim et al., 2003, 2007). Polyclonal anti-HSP90 antibodies were generated in rabbits and were diluted 1:5,000 for detection. Nuclear or cytosolic fractions were generated with/without the NP40 detergent in protein extraction buffer or using the CelLytic PN-Plant Nuclei Isolation Kit (Sigma-Aldrich).

### Flowering Time Measurement

To measure flowering time, seeds were sown on soil following stratification (4°C for 2 days). Plants were grown under LD condition (16L/8D). Flowering time was measured by counting the number of rosette leaves when the first flower opened.

### Chromatin Immunoprecipitation and Quantitative PCR (ChIP-qPCR)

Approximately 2 g of the third and fourth leaf tissue harvested from 5 weeks old plants was fixed in 1% formaldehyde and crosslinked under vacuum for 15 min. A final concentration of 0.25 M glycine was used to quench the formaldehyde for 5 min under vacuum. After washing twice with cold deionized water, the tissue was ground in liquid N2, and chromatin was extracted as described previously (Zhu et al., 2013). ChIP assays were performed using the ChIP Kit-Plants (ab117137, Abcam). Nuclei extraction and chromatin disruption were performed using a sonicator with 10 cycles on ice (10 s each at amplitude 25%/50 s cooling). Chromatin was immunoprecipitated using anti-GFP antibody (ab290, Abcam)-bound assay plate for 90 min. The resulting immunoprecipitated DNA was subjected to qPCR to examine the enrichment of target genes using primers listed in Supplementary Table 1. Three biological replicates were performed.

### Chemical Induction of GI

To perform methoxyfenozide (MOF) treatments, inducible GI-GFP (*VGE:GI-GFP*) plants were grown in soil under LD conditions. After 3 weeks, *VGE:GI-GFP* plants were soaked in dimethyl sulfoxide (DMSO; mock control) or 50 μM MOF for 6 h. After 2 weeks, samples were collected for analyzing leaf senescence. Six biological replicates were performed.

## Results

### Leaf Senescence Is Altered by Day Length

In *Arabidopsis*, a facultative LD plant, flowering is induced by LDs (Corbesier et al., 1996). The circadian clock regulates various developmental processes including seedling growth and flowering time, depending on the photoperiod (de Montaigu et al., 2010). In Aspen tree, SD photoperiod acts as the main trigger of autumn senescence (Fracheboud et al., 2009). Therefore, we first examined whether leaf senescence in *Arabidopsis* is also photoperiod dependent, similar to flowering. We used the third and fourth rosette leaves for all experiments because both leaves represent a different age status (Zentgraf et al., 2004). Three-weeks-old wild-type plants grown under the LD condition were transferred to either LD or SD photoperiod to avoid any developmental issues, and leaf yellowing was used as a visual indicator of leaf senescence. Leaves of wild-type plants started yellowing at 28 days after emergence (DAE) under the LD photoperiod but did not start yellowing until 40 DAE under the SD photoperiod (Supplementary Figure 1A). Other senescence indicators, including Fv/Fm and chlorophyll content, were consistent with the leaf yellowing phenotype (Supplementary Figures 1B,C). These results suggest that leaf senescence is induced by LD photoperiod, similar to flowering, in *Arabidopsis*.

### GI Positively Regulates Leaf Senescence

**FIGURE 1:**
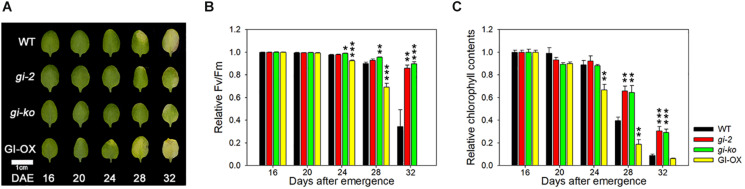
GI positively regulates leaf senescence in *Arabidopsis*. **(A)** Photographs showing yellowing of wild-type (Col-0), *gi-2*, *gi-ko*, and *CsV:GI-GFP* leaves at the indicated days after emergence (DAE). The third and fourth rosette leaves were used in this experiment. Scale bar: 1 cm. **(B,C)** Photochemical efficiency (Fv/Fm) **(B)** and chlorophyll content **(C)** of leaves of the indicated genotypes. Data are presented as mean ± standard error of mean (SEM). Six biological replicates were performed. Asterisks indicate significant differences (*p < 0.05, **p < 0.01, ***p < 0.005; Student’s *t*-test).

GI is known as a mediator between the circadian clock and photoperiod-dependent flowering (Park et al., 1999). The circadian clock senses the photoperiod and relays it to GI to regulate flowering (Sawa et al., 2007). While *gi* mutants exhibit late flowering, overexpression of GI rescues the late flowering phenotype of *gi* mutants (Sawa and Kay, 2011). Previously, *gi-2* mutant showed the delayed leaf senescence phenotype in an age-dependent manner (Kim et al., 2018). In this study, we tested the leaf senescence phenotype of another *gi* mutant, *gi-ko* (*gi-201*), and examined the effect of GI overexpression in the *gi-2* mutant. Under the LD photoperiod, the *gi-ko* mutant maintained green leaves, similar to the *gi-2* mutant, until 32 DAE compared with the wild type. However, *gi-2* mutant plants overexpressing GI showed yellowing of leaf tips at 24 DAE, with greater yellowing at 28 DAE (Figure 1A). The values of Fv/Fm and chlorophyll contents were consistent with the leaf yellowing phenotype (Figures 1B,C). This result indicates that GI positively regulates leaf senescence, similar to flowering.

To further test the direct influence of GI on leaf senescence, we used MOF-inducible *VGE:GI-GFP* transgenic plants. After 3 weeks of growth under LD photoperiod, we soaked the transgenic plants in DMSO (control) or 50 μM MOF for 6 h. Leaf yellowing was significantly greater in the leaves of MOF-treated *plants* than in the leaves of control plants (Supplementary Figure 2A). The Fv/Fm values and chlorophyll contents were consistent with the leaf yellowing phenotype (Supplementary Figures 2B,C). These results suggest that GI directly affects leaf senescence in *Arabidopsis.*

### GI Controls Leaf Senescence in a Location-Dependent Manner

GI differentially controls the circadian clock and flowering, depending on its subcellular localization. While both nuclear and cytosolic GI regulate the circadian rhythm, only nuclear GI promotes flowering by binding to *CO* or *FT* promoter (Kim et al., 2013a). In the nucleus, ELF4 directly interacts with GI, thus inhibiting its binding to the target gene promoter (Kim et al., 2013b). Here, we hypothesized that GI controls leaf senescence in a manner similar to how it controls flowering time. To test this hypothesis, we first tested the effect of the location of GI in the cell on leaf senescence. We used transgenic lines expressing *CsV:GI-GFP-NLS* or *CsV:GI-GFP-NES* in the *gi-2* mutant background, in which the GI-GFP fusion protein is preferentially localized to the nucleus or cytosol, respectively (Supplementary Figure 3A). We then examined alterations of circadian physiology, such as flowering time in these transgenic plants. *CsV:GI-GFP* complemented the flowering physiological lesions altered by the *gi-2* mutation (Supplementary Figure 3B). *CsV:GI-GFP-NLS* and *CsV:GI-GFP-NES* differentially complemented the circadian alterations: the late flowering phenotype of *gi-2* mutants was complemented by *CsV:GI-GFP-NLS* but not by *CsV:GI-GFP-NES* (Supplementary Figure 3B).

**FIGURE 2:**
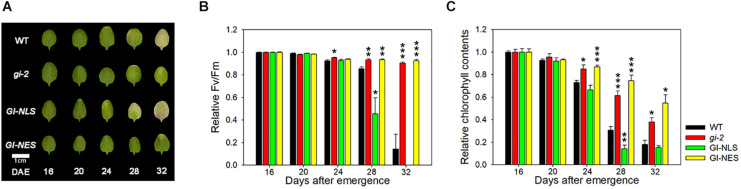
Subcellular localization of GI determines its effect on leaf senescence. **(A)** Photographs showing yellowing of wild-type (Col-0), *gi-2*, *CsV:GI-GFP-NLS*, and *CsV:GI-GFP-NES* leaves. The third and fourth rosette leaves were used for this experiment. DAE, days after emergence. Scale bar: 0.5 cm. **(B,C)** Fv/Fm ratio **(B)** and chlorophyll content **(C)** of leaves of the indicated genotypes at the indicated time points. Data are presented as the mean ± SEM. Six biological replicates were performed. Asterisks indicate significant differences (*p < 0.05, **p < 0.01, ***p < 0.005; Student’s *t*-test).

In the LD condition, *CsV:GI-GFP-NLS* plants showed an early leaf yellowing phenotype compared with wild-type plants, whereas *CsV:GI-GFP-NES* plants showed no leaf yellowing, similar to the *gi-2* mutant (Figure 2A). Compared with the wild type, the Fv/Fm value of *CsV:GI-GFP-NLS* leaves was clearly reduced at 28 DAE, whereas that of *CsV:GI-GFP-NES* leaves was maintained until 32 DAE, similar to *gi-2* mutant leaves (Figure 2B). Additionally, the chlorophyll content of *CsV:GI-GFP-NLS* leaves rescued the *gi-2* mutant phenotype, whereas that of *CsV:GI-GFP-NES* leaves did not (Figure 2C), indicating that only nuclear-localized GI rescues the delayed senescence phenotype of the *gi* mutant. Taken together, these results suggest that the subcellular localization of GI determines leaf senescence, similar to flowering.

### ELF4 Negatively Regulates the Role of GI in Leaf Senescence

**FIGURE 3:**
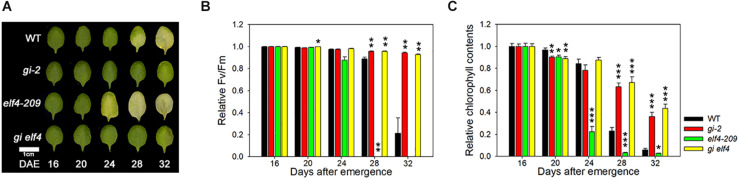
The *gi-2* mutant allele is epistatic to *elf4-209* in the leaf senescence pathway. **(A)** Photographs showing yellowing of leaves of wild-type (Col), *gi-2*, *elf4-209*, and *gi elf4* plants at the indicated time points. DAE, days after emergence. The third and fourth rosette leaves were used for this experiment. Scale bar: 1 cm. **(B,C)** Fv/Fm ratio **(B)** and chlorophyll content **(C)** in leaves of the indicated genotypes at the indicated time points. Data are presented as mean ± SEM. Six biological replicates were performed. Asterisks indicate significant differences (*p < 0.05, **p < 0.01, ***p < 0.005; Student’s *t*-test).

In the nucleus, ELF4 physically interacts with and inhibits the binding of GI to the *CO* promoter to induce flowering (Kim et al., 2013b). The *gi* mutant allele is epistatic to *elf4* in photoperiod-dependent flowering time regulation (Kim et al., 2012). Thus, we first tested whether *gi* and *elf4* share a similar hierarchy in the leaf senescence pathway. The *elf4-209* single mutant showed early senescence, whereas the *gi-2* mutant showed delayed senescence (Kim et al., 2018). The timing of leaf yellowing in the *gi elf4* double mutant was similar to that of the *gi* single mutant under LDs (Figure 3A). We further quantitatively evaluated the senescence time of all mutant combinations by measuring Fv/Fm values and chlorophyll contents with leaf aging. The timing of leaf senescence in the *gi elf4* double mutant was similar to that in the *gi* single mutant but not to that in the *elf4* single mutant (Figures 3B,C). These results indicate that like the flowering phenotype, the early senescence phenotype of the *elf4* single mutant is masked by the *gi* mutant.

**FIGURE 4:**
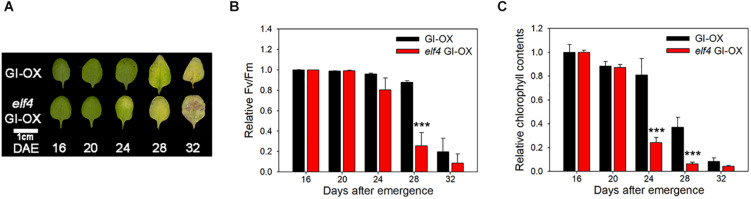
ELF4 alters the leaf senescence phenotype of *GI* overexpression (*GI-OX*) plants. **(A)** Photographs showing yellowing of *CsV:GI-GFP* (*GI-OX*) and *elf4 CsV:GI-GFP* (*elf4 GI-OX*) leaves at the indicated time points. DAE, days after emergence. The third and fourth rosette leaves were used for this experiment. Scale bar: 1 cm. **(B,C)** Fv/Fm ratio **(B)** and chlorophyll content **(C)** of leaves of the indicated genotypes at the indicated time points. Data are presented as mean ± SEM. Six biological replicates were performed. Asterisks indicate significant differences (***p < 0.005; Student’s *t*-test).

Next, we tested whether ELF4 negatively regulates the effect of GI on leaf senescence, similar to flowering (Kim et al., 2013b). We compared the leaf senescence phenotype of wild-type and *elf4* mutant plants overexpressing GI. We found that early senescence of GI-overexpressing plants was more accelerated in the *elf4* mutant. Leaf yellowing progressed at a faster rate in GI-OX/*elf4* plants than in GI-OX/Col-0 plants (Figure 4A). The Fv/Fm values and chlorophyll contents of GI-OX/*elf4* and GI-OX/Col-0 plants were consistent with their leaf yellowing phenotypes (Figures 4B,C). This result indicates that ELF4-free GI controls leaf senescence in a similar manner as it controls flowering.

### GI Positively Regulates Leaf Senescence by Activating ORE1

**FIGURE 5:**
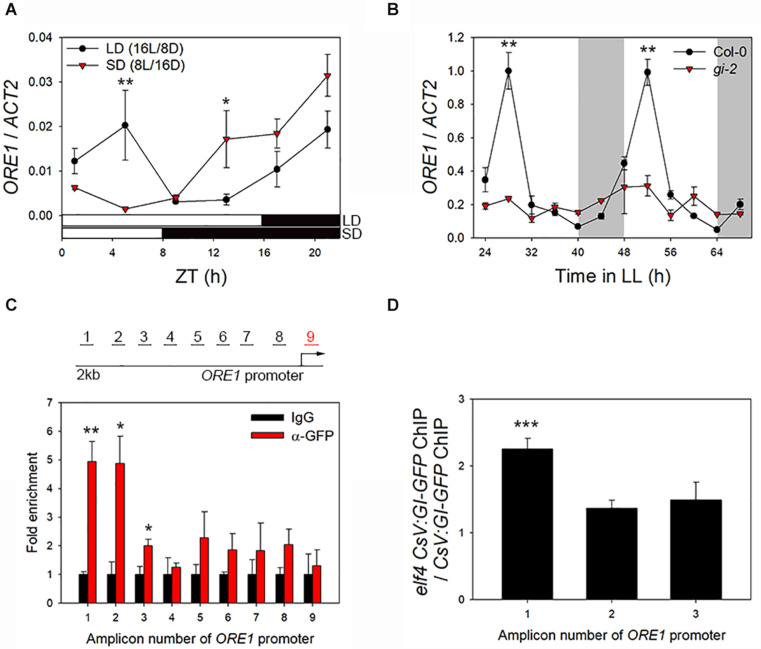
GI binds to *ORE1* promoter and activates its expression. **(A)** Diurnal expression of *ORE1* under long-day (LD; 16 h light/8 h dark) and short-day (SD; 8 h light/16 h dark) photoperiod. **(B)** Abundance of *ORE1* mRNA in wild-type and *gi-2* mutant leaves under continuous light (LL). White bars indicate subjective day, and gray shading indicates subjective night. **(C)** *ORE1* promoter-binding affinity of GI in *CsV:GI-GFP* transgenic plants. Schematic representation of amplicons 1–9 of the *ORE1* promoter amplified in the chromatin immunoprecipitation-quantitative PCR (ChIP-qPCR) assay are indicated. Amplicon 9 was used as an internal control. Leaves were harvested at 12 h after light on. Fold enrichment indicates the ratio of chromatin immunoprecipitated using anti-GFP antibody to that immunoprecipitated using anti-IgG antibody. Asterisks indicate statistically significant difference compared with the IgG control. **(D)** *ORE1* promoter-binding activity of GI in the *elf4* mutant relative to that in the wild type at amplicons 1, 2, and 3. Three biological replicates were performed. Asterisks indicate significant differences (*p < 0.05, **p < 0.01, ***p < 0.005; Student’s *t*-test).

ORE1 promotes senescence in *Arabidopsis* (Kim et al., 2009). We previously reported that ORE1 is under the control of the circadian clock, and the clock component, PRR9, binds to the *ORE1* promoter to induce leaf senescence (Kim et al., 2018). Additionally, we suggested that GI acts upstream of *ORE1* (Kim et al., 2018). Here, we hypothesized that GI controls leaf senescence by inducing *ORE1* expression. To test this hypothesis, we first examined the effect of the photoperiod on *ORE1* expression. To avoid the difference in plant growth between SD and LD conditions, we transferred wild-type plants to either SD or LD photoperiod after 3 weeks of growth under LDs. *ORE1* expression showed two peaks under the LD condition, morning peak (ZT4) and evening peak (ZT20) (Figure 5A). However, the morning peak of *ORE1* was absent under the SD condition, despite higher expression level of *ORE1* during the dark phase under the SD photoperiod than under the LD photoperiod (Figure 5A). We previously showed that in plants entrained under the LD condition and then moved to continuous light, *ORE1* exhibits one peak in the morning, which is controlled by the circadian clock (Kim et al., 2018). These results suggest that the morning peak of *ORE1* is important for the circadian clock-mediated regulation of leaf senescence. Thus, we tested expression of *ORE1* in wild-type and *gi-2* mutant plants under continuous light. The expression of *ORE1* was significantly reduced in the *gi-2* mutant at 28 h and 52 h in LL compared with the wild-type (Figure 5B). To further confirm the effect of GI on the expression of *ORE1*, we used *VGE:GI-GFP* transgenic plants. Ten-days-old *VGE:GI-GFP* seedlings grown in 1/2 B5 media were transferred to DMSO (control) or MOF-containing media at ZT10 (GI peak time). After 24 h, MOF treatment showed ∼2-fold increase in *ORE1* transcript level compared with the control (Supplementary Figure 4). These results suggest that GI acts as a direct transcriptional activator of *ORE1*.

In yeast (Saccharomyces cerevisiae), GI binds to the *ORE1* promoter (Kim et al., 2018). Thus, we tested the direct binding of GI to the *ORE1* promoter in *Arabidopsis* leaves by ChIP-qPCR. Leaves of GI-OX plants at ZT12 (GI protein peak time) were used for ChIP, and the binding of GI to eight different test regions (amplicons 1–8) or to an internal control region (amplicon 9) of the *ORE1* promoter (∼2 kb upstream of the transcription start site) was tested by qPCR to screen the GI binding region on *ORE1* promoter (Figure 5C, upper diagram). GI showed significantly greater binding affinity toward amplicons 1, 2, and 3 of the *ORE1* promoter than the negative control (anti-IgG antibody) (Figure 5C). Enrichment of the internal control region (amplicon 9) showed no significant difference between the anti-IgG antibody (control) and anti-GFP antibody samples. These results suggest that GI regulates leaf senescence by directly binding to the *ORE1* promoter and activating its expression. Previously, we showed that ELF4 inhibits the binding of GI to the *CO* promoter (Kim et al., 2013b). Therefore, we tested whether ELF4 affects the activity of GI as a transcriptional activator of *ORE1* by conducting ChIP assays using CsV:GI-GFP and *elf4* CsV:GI-GFP leaves. GI-GFP was enriched > twofold in the *elf4* mutant background compared with the wild type at amplicon 1, but no significant difference was detected at the other amplicons (Figure 5D). These results suggest that nuclear-localized ELF4 and GI proteins together regulate leaf senescence. Thus, the regulation of leaf senescence in *Arabidopsis* is similar to that of flowering.

## Discussion

**FIGURE 6:**
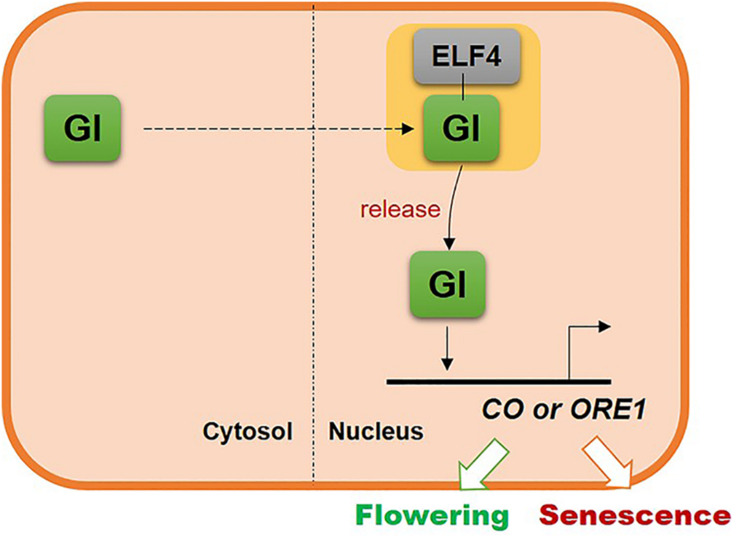
Schematic illustrating the common molecular mechanism underlying the regulation of flowering and leaf senescence by GI.

The results of this study suggest that GI, one of the clock components, controls leaf senescence in *Arabidopsis* via the same regulatory mechanism as that used to control flowering. The location of GI in the cell is critical for regulating the timing of leaf senescence, similar to flowering. In the nucleus, GI is sequestered by ELF4 and then released at the proper developmental stage to induce *CO* or *ORE1* expression and consequently flowering or senescence, respectively. These results suggest that plants use a common regulatory mechanism to couple flowering with senescence (Figure 6).

Depending on the subcellular localization, a single protein can regulate several signaling pathways and developmental processes such as flowering and senescence. These two developmental stages have a common purpose in the plant life cycle; flowering switches the transition from the vegetative growth phase to the reproductive phase, while senescence remobilizes the nutrients to newly developing leaves or seeds. This implies that flowering and senescence are closely connected and need similar regulatory process to maximize fitness. GI, a key clock regulator, connects both these developmental processes successfully.

GI is involved in multiple processes including redox signaling in *Arabidopsis* (Mishra and Panigrahi, 2015). Deficiency of GI results in increased tolerance to redox signals. Chlorophyll accumulation is higher in *gi* mutants than in the wild-type under oxidative stress conditions (Kurepa et al., 1998). However, the molecular mechanism that links the cellular localization of GI with redox signaling for plant developments including flowering and senescence is unknown. Further research is required to elucidate this molecular mechanism.

In this paper, we suggest that GI protein directly binds to *ORE1* promoter to induce *ORE1* expression. Additional explanation is required for this process since there is a significant temporal distance between the expression timing of these two molecules. The expression of GI protein peaks around ZT12, while *ORE1* gene expression peaks around ZT4. We searched for a similar case in *Arabidopsis* circadian clock, and found that RVE8, which peaks in the morning, is known to directly activate evening genes, including *PRR5* and *TOC1* (Rawat et al., 2011). However, we cannot provide any results in this paper to suggest an underlying mechanism for the temporal gap between GI abundance and *ORE1* expression. It would be an interesting topic for further study.

ORE1, a key senescence regulator, is controlled by the circadian clock. The role of ORE1 in senescence is similar to that of CO in flowering. Like *CO*, *ORE1* shows two peaks under the LD photoperiod, and its peak during the subjective day disappears under the SD photoperiod. This indicates that day-peaks of *ORE1* and *CO* expression are equally important for inducing senescence and flowering, respectively. These results suggest that GI regulates flowering and senescence in *Arabidopsis* using different partner but via the same mechanism. It thus appears that GI was co-opted during evolution to coordinate the reproductive phase and senescence process, which provides important insight into the concerted evolution of reproduction and senescence.

## Data Availability Statement

The original contributions presented in the study are included in the article/Supplementary Materials, further inquiries can be directed to the corresponding author/s.

## Author Contributions

HK and HGN designed all the experiments, prepared the figures, and wrote the manuscript. HK, SJP, and YK performed all the experiments. All authors contributed to the article and approved the submitted version.

## Conflict of Interest

The authors declare that the research was conducted in the absence of any commercial or financial relationships that could be construed as a potential conflict of interest.
