# Supplementary material for: Subcellular Localization of GIGANTEA Regulates the Timing of Leaf Senescence and Flowering in Arabidopsis
Source: Front Plant Sci. 2020 Nov 19;11:589707. doi: 10.3389/fpls.2020.589707 (PMC7710859; doi:10.3389/fpls.2020.589707)
Supplement: Supplementary file 1 [file Data_Sheet_1.docx]

Supplementary Material

# Supplementary Figures and Tables

## Supplementary Figures


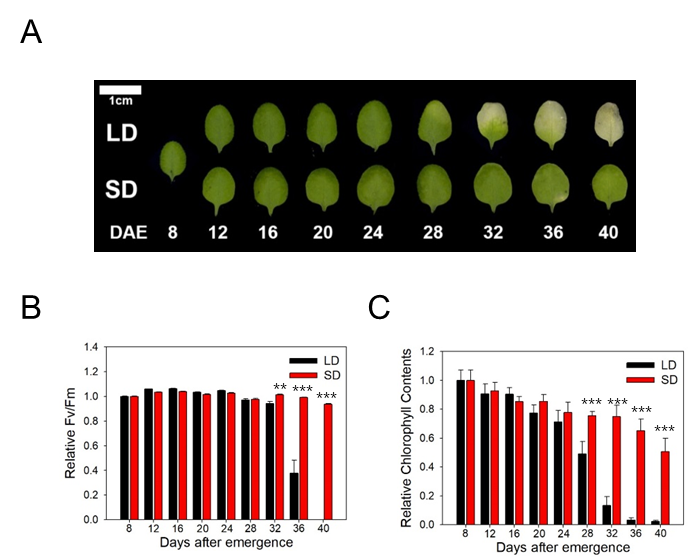


**Supplementary Figure 1.** Photoperiod affects the timing of leaf senescence. (A) Photographs showing yellowing of wild-type leaves under long-day (LD; 16 h light/8 -h dark) and short-day (SD; 8 h light/16 h dark) photoperiods. The third and fourth rosette leaves were used in this experiment. DAE, days after emergence. Scale bar: 1 cm. (B and C). Analysis of photochemical efficiency (Fv/Fm) (B) and chlorophyll contents (C) of leaves under SD and LD photoperiods. Data are presented as mean ± standard error of mean (SEM). Six biological replicates were performed. Asterisks indicate significant differences (***p* < 0.01,****p* < 0.005; Student’s *t*-test).

#
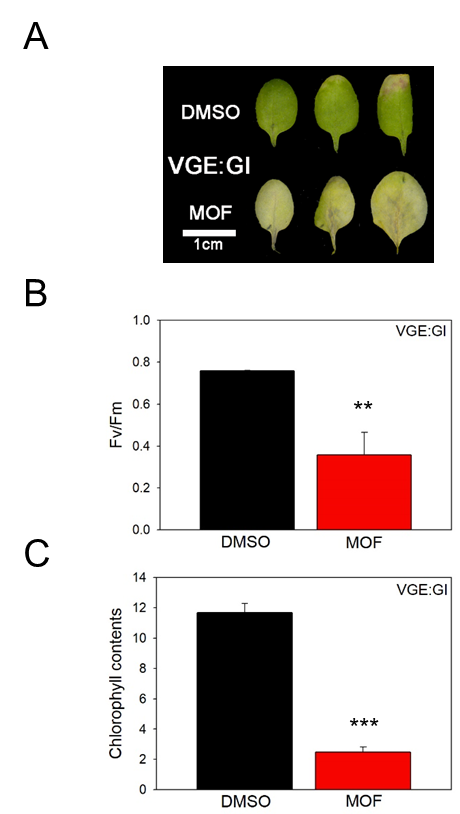


**Supplementary Figure 2.** Transient induction of *GIGANTEA* (*GI*) induces leaf senescence. (A) Photographs showing yellowing of *VGE::GI-GFP* leaves after treatment with dimethyl sulfoxide (DMSO) or methoxyfenozide (MOF). The third and fourth rosette leaves were used for this experiment. Scale bar: 1 cm. (B and C) Fv/Fm ratio (B) and chlorophyll contents (C) of leaves treated with DMSO or MOF. Six biological replicates were performed. Data are presented as mean ± SEM. Asterisks indicate significant differences (***p* < 0.01,****p* < 0.005; Student’s *t*-test).


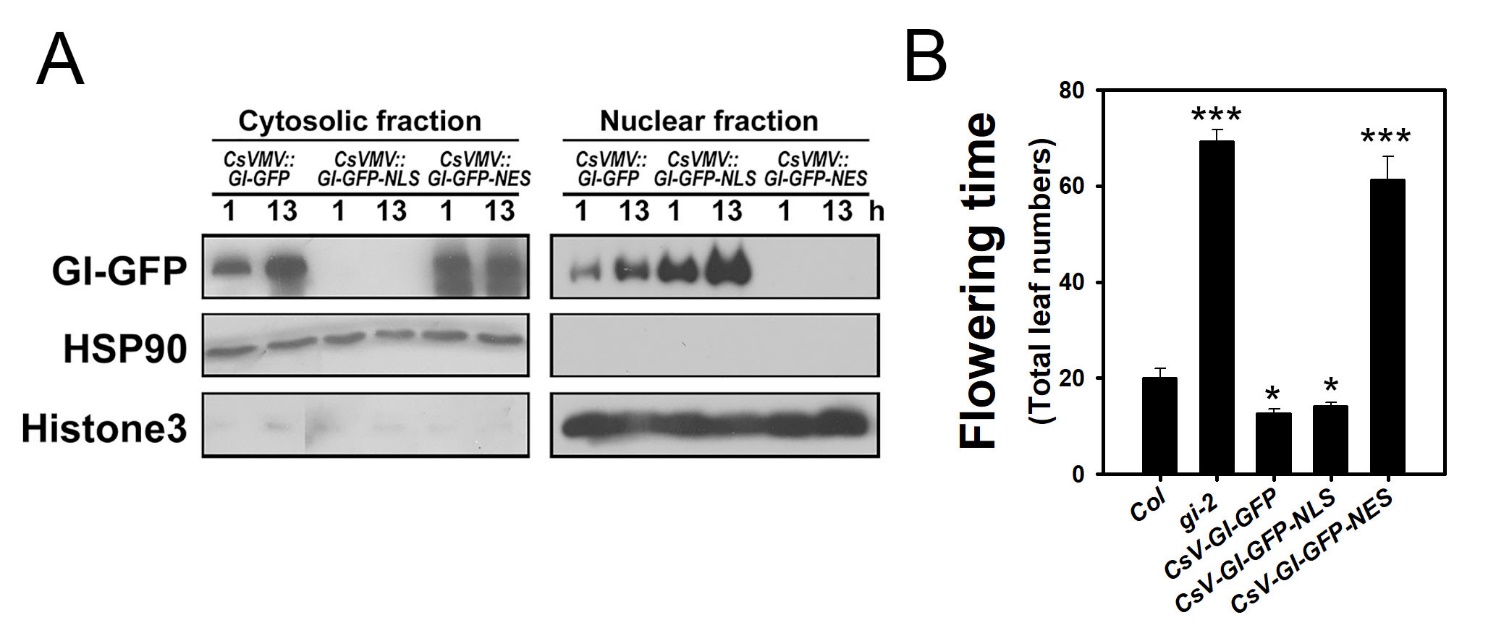


**Supplementary Figure 3.** Nucleus- and cytosol-localized GI have differential roles in controlling flowering in *CsV::GI-GFP-NLS* and *CsV::GI-GFP-NES* transgenic plants. (A) GI protein localization in transgenic *CsV::GI-GFP*, *CsV::GI-GFP-NLS* and *CsV::GI-GFP-NES* plants. GI proteins were detected in 10-day-old seedlings grown under 12L/12D condition at ZT1 and ZT13. HSP90 and H3 were used as cytosolic and nuclear marker, respectively. (B) Flowering time of wild-type, *gi-2*, *CsV::GI-GFP*, *CsV::GI-GFP-NLS*, and *CsV::GI-GFP-NES* plants, indicated by the total number of leaves at the time of first flower opening under LD conditions. Data are presented as mean ± SEM. Asterisks indicate significant differences (**p* < 0.05,****p* < 0.005; Student’s t-test).


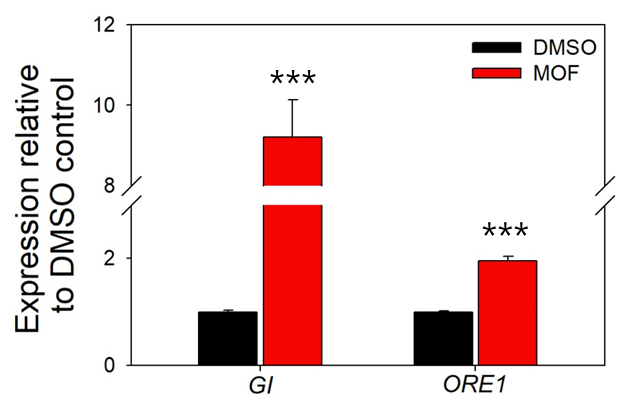


**Supplementary Figure 4.** Comparison of *GI* and *ORE1* transcript levels in DMSO or MOF-treated *VGE::GI-GFP* transgenic plants. Ten-day-old *VGE::GI-GFP* seedlings were treated with MOF at ZT10 (*GI* peak time), and samples were harvested after 24 h. Data are presented as mean ± SEM. Three biological replicates were performed. Asterisks indicate significant differences (****p* < 0.005; Student’s *t*-test).

## Supplementary Tables

**Supplementary Table 1.** List of primers used in this study.

| **Target gene/amplicon** | **Forward primer (5'→3')** | **Reverse primer (5'→3')** |
| --- | --- | --- |
| *Quantitative real-time PCR (qRT-PCR)* | | |
| *ORE1* | GTGGGTATGAAGAAAACTTTGG | TTCGTTCTTAGCTGTTTGGGG |
| *GI* | AATTCAGCACGCGCCTATTG | GTTGCTTCTGCTGCAGGAACTT |
| *Chromatin immune precipitation-quantitative PCR (ChIP-qPCR)* | | |
| Amplicon 1 | ATCTCTATTAACGGTTACCATC | TTTCAAGACATAGCGTGTG |
| Amplicon 2 | CTAGAGTCTTACATTCTCTTTGG | CGTATGTATATGGCTGGAATG |
| Amplicon 3 | GACAACCTCGTATGAACAAA | CCATGTGAAGGTGGTAATG |
| Amplicon 4 | GTAGACACTGGAATAAGACAAG | CGGTACGGGTCACTATTAT |
| Amplicon 5 | TCTCTTCAAACCATTCACAC | CCATTCTTTATCTCATTATCAACG |
| Amplicon 6 | AGGGAGTTACAGGTGAAG | AGAAGATGCGTAGATTGTTAG |
| Amplicon 7 | CGCGAAACCTCATGTAT | TCGTATCAAGTTCTTCTTTAG |
| Amplicon 8 | TATAGAGAGGAGCTTCGTTG | TGTTGTTGTTGTGTTTGTTG |
| Amplicon 9 | CCATGTAAGTTTCACACCTTATC | GGTCTCTCACACAGAAGAAATA |
